# CCN3 modulates bone turnover and is a novel regulator of skeletal metastasis

**DOI:** 10.1007/s12079-012-0161-7

**Published:** 2012-03-18

**Authors:** Véronique Ouellet, Peter M. Siegel

**Affiliations:** 1 Goodman Cancer Research Centre, McGill University, 1160 Pine Avenue West, Room 513, Montreal, Quebec Canada H3A 1A3; 2 Departments of Anatomy and Cell Biology, Biochemistry and Medicine, McGill University, Montreal, Quebec Canada

**Keywords:** Bone primary cancer, Bone metastasis, Breast cancer, CCN3, Osteoblast, Osteoclast

## Abstract

The CCN family of proteins is composed of six secreted proteins (CCN1-6), which are grouped together based on their structural similarity. These matricellular proteins are involved in a large spectrum of biological processes, ranging from development to disease. In this review, we focus on CCN3, a founding member of this family, and its role in regulating cells within the bone microenvironment. CCN3 impairs normal osteoblast differentiation through multiple mechanisms, which include the neutralization of pro-osteoblastogenic stimuli such as BMP and Wnt family signals or the activation of pathways that suppress osteoblastogenesis, such as Notch. In contrast, CCN3 is known to promote chondrocyte differentiation. Given these functions, it is not surprising that CCN3 has been implicated in the progression of primary bone cancers such as osteosarcoma, Ewing’s sarcoma and chondrosarcoma. More recently, emerging evidence suggests that CCN3 may also influence the ability of metastatic cancers to colonize and grow in bone.

## The CCN family of proteins

The CCN family of proteins emerged as an integrated entity in 2003, when a unified nomenclature was adopted by the scientific community to reduce growing confusion in the literature (Brigstock et al. 2003). The first letter from the three founding members, which include **C**yr 61 (cysteine rich 61), **C**TGF (connective tissue growth factor) and **N**ov (nephroblastoma overexpressed), was used to create the acronym CCN(Perbal and Takigawa 2005). These three proteins became known as CCN1, CCN2 and CCN3, respectively, and three additional members were also identified at that time, including CCN4 (WISP-1), CCN5 (WISP-2) and CCN6 (WISP-3). Based on a similar domain structure, these six members have been grouped into the CCN family of matricellular proteins.

## CCN3, the nephroblastoma overexpressed gene

As its original name suggests, CCN3 was first discovered as a gene that was overexpressed in a myeloblastosis-associated virus (MAV-1)-induced nephroblastoma that developed in chicken (Joliot et al. 1992). CCN3 is a cysteine-rich secretory protein that associates with components of the extracellular matrix. Since the discovery of this matricellular protein, CCN3 has been implicated in an increasing number of diverse biological processes including proliferation, differentiation and angiogenesis as well as pathological conditions such as fibrosis and cancer (Perbal 2001; Brigstock 2003; Leask and Abraham 2006; Perbal 2006; Chen and Lau 2009; McCallum and Irvine 2009; Zuo et al. 2010; Kular et al. 2011). This diversity of CCN3 function is not surprising given the modular nature of CCN family members, which contains four distinct domains that enable binding to numerous protein partners. Layered on top of this large protein interaction network, is the growing appreciation for the importance of differential splicing and post-translational modifications, such as glycosylation and proteolytic cleavage, which can greatly influence CCN3 activity. Emerging evidence positions CCN3 as an important modulator of cancer progression and metastasis. Thus, we will focus this review specifically on these aspects of CCN3 biology as they relate to normal bone turnover, the growth of primary bone tumors and cancers that metastasize to bone.

## The modular structure of CCN3

**Fig. 1:**
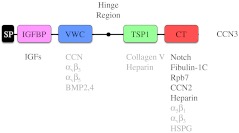
Schematic diagram depicting the modular domains of CCN3. CCN3 shares a similar overall structural organization with the remaining CCN family members, including a secretory signal peptide (SP), an insulin-like growth factor binding protein domain (IGFBP), a von Willebrand factor type C domain (VWC), a Thrombospondin-1 type repeat (TSP-1) and a carboxyl-terminal domain (CT) that contains a cysteine knot. Known binding proteins are listed in *black* underneath the particular domain through which they interact with CCN3. Proteins denoted in *grey* are those for which the precise CCN3 domain responsible for the interaction remains unknown, but has been defined in other CCN family members. IGFs: Insulin-like Growth Factors, BMP: Bone Morphogenetic Protein; Rbp7: Retinol Binding Protein 7; HSPG: Heparan Sulfate Proteoglycan

With one exception, all CCN family members possess an N-terminal signal peptide (SP) followed by four modular domains with homology to an insulin-like growth factor binding protein (IGFBP) domain, a von Willebrand factor type C domain (VWC), a Thrombospondin-1 type repeat (TSP-1) and a C-terminal region (CT) containing a cysteine knot structure. CCN5 is the only family member that deviates from this overall domain organization due to the absence of the CT domain (Pennica et al. 1998; Zhang et al. 1998). In each case, the VWC and TSP-1 domains are separated by a variable hinge region (Fig. 1).

### IGFBP domain

The importance of this module as a functional Insulin-like Growth Factors (IGF) binding domain in CCN proteins, and its biological significance, remains unclear. Three independent investigations have shown that CCN3 protein lack or exhibit very low binding affinities for IGF (Kim et al. 1997; Burren et al. 1999; Vorwerk et al. 2002). The core members of the IGFBP family contain both an N- and C-terminal IGF binding domain, which act in concert to promote high affinity ligand binding (Payet et al. 2003). In contrast, CCN proteins possess a single N-terminal IGFBP domain. Thus, the 100-fold reduction in IGF binding exhibited by CCN proteins, relative to IGFBPs, was postulated to be due to the lack of this C-terminal IGF binding domain. However, a chimeric protein created by substituting the N-terminal IGFBP domain of IGFBP-3 with the N-terminal IGFBP domain of CCN3 displayed very weak binding to IGFs similar to that observed for CCN3. These data argue that the CCN3 IGFBP domain is non-functional for binding IGFs, even in the context of an N- and C-terminal IGFBP domain configuration (Yan et al. 2006).

### VWC domain

This domain, also known as chordin-like cysteine rich repeat, is commonly found in extracellular matrix proteins. The VWC module was shown to be important for the ability of CCN family members to bind Bone Morphogenetic Protein (BMP) and Transforming Growth Factor beta (TGF-β) (Abreu et al. 2002). In addition, it has been suggested that this domain plays a role in oligomerization with members of the CCN family and a diverse array of interacting proteins (Voorberg et al. 1991; Brigstock 1999; Perbal 2001).

### Hinge region

This region connects the VWC domain to the TSP-1 like domain and is believed to be highly susceptible to proteolytic processing. It was recently shown that Kallikrein related peptidase 12 preferentially cleaved CCN1 within the hinge region, although this protease, when incubated *in vitro* with different recombinant CCN proteins, including CCN3 displayed multiple target sites for cleavage (Guillon-Munos et al. 2011). Additional proteins, including Elastase, Plasmin and Matrix Metalloproteases (MMP-1, MMP-2, MMP-3, MMP-7, MMP-9 and MMP-13), have all been described to cleave CCN family proteins *in vitro* (Brigstock et al. 1997; Hashimoto et al. 2002; de Winter et al. 2008). While the relationship between processing events that have been described *in vitro* to those that occur naturally need to be further clarified, these cleaved CCN variants and/or their ratio to the full length protein are thought to significantly impact the functions of CCN family members.

### TSP-1 domain

This domain is commonly found in a variety of extracellular matrix proteins. Within the CCN family, this region functions as a protein interaction domain that facilitates binding to Vascular Endothelial Growth Factor (VEGF) (Inoki et al. 2002), integrin receptors and Low-density Related Lipoprotein (LRP) (Gao and Brigstock 2003). These various interactions have been shown to be important for the ability of CCN family members to control tumor angiogenesis and breast cancer cell adhesion (Ellis et al. 2003; Gao and Brigstock 2003; Lin et al. 2005; Tong and Brigstock 2006)(reviewed in (Holbourn et al. 2009)).

### CT domain

The C-terminal module was named based on the presence of a cysteine knot structure and is known to bind a variety of proteins such as platelet-derived growth factor, nerve growth factor, TGF-β, Heparin, Heparan Sulfate Proteoglycans (HSPGs), Notch1 and Fibulin-1C (Brigstock et al. 1997; Kireeva et al. 1997; Chevalier et al. 1998; Perbal et al. 1999; Sakamoto et al. 2002; Ball et al. 2003). In addition, this module promotes homo- or heterodimerization of CCN proteins (Perbal et al. 1999; Lazar et al. 2007). The CT domain also contains a nuclear localization signal, which has been implicated in promoting interactions between CCN proteins with transcription regulatory proteins (Mahony et al. 1999; Perbal 1999; Planque et al. 2006). The functional implications of nuclear functions are not yet fully elucidated.

Thus, it is clear that the modular domain structure of CCN3 permits diverse protein interactions with members of the CCN family, which may explain why these proteins elicit different cellular responses depending on the cellular context.

## CCN3 isoforms

The complexity surrounding CCN3 is, in part, related to the different alternative splice variants and post-translational modifications that alter CCN3 biological function (Perbal 2009). Several variants of CCN3 have been described in the literature; however, their origins are not well described. Thus, it is a challenge to determine whether these variants result from alternate splicing or proteolytic cleavage or other post-translational modifications. The human CCN3 is composed of 357 amino acids with a predicted size of 44 kDa. It possesses two putative N-glycosylation sites, one at position 97 (NQTG) and the second at 280 (NCTS), which results in a secreted CCN3 protein with an apparent molecular weight of 54 kDa (Chevalier et al. 1998). Thus, higher molecular weight CCN3 isoforms result from post-translational glycosylation and the lower molecular weight species are likely derived from alternative splicing or proteolytic cleavage.

The first CCN3 variant to be described was an amino-terminal truncation that resulted from integration of the MAV virus, which removed the signal peptide and the IGFBP domain, leaving only the lasts three modules (VWC, TSP-1 and CT) (Joliot et al. 1992). This CCN3 variant was expressed in chicken embryo fibroblasts and exhibited transforming properties. While it is unclear whether this isoform occurs naturally, it represents the first evidence of a truncated form of CCN3 that retains biological activity.

### Alternative splicing

Different studies have reported CCN3 variants that most likely arise from alternative splicing (reviewed in (Perbal 2009)). Although consensus sequences defining alternative splice sites have yet to be described for CCN3, they have been identified in CCN1 (Martinerie et al. 1997; Leng et al. 2002; Hirschfeld et al. 2009) and CCN4 (Tanaka et al. 2001, 2003; Cervello et al. 2004; Yanagita et al. 2007). However, the use of domain specific antibodies has facilitated the identification of two CCN3 isoforms. One lacks the TSP-1 domain and was reported to be localized in the nucleus of kidney cancers (Subramaniam et al. 2008). The other isoform of CCN3, devoid of the VWC module, was detected in Ewing sarcoma cancer cells as well as in developing mouse tissues (Heath et al. 2008; Perbal et al. 2009). While the presence of these CCN3 isoforms is intriguing, their precise biological roles have yet to be elucidated.

### Proteolytic processing

Numerous publications have reported proteolytically cleaved forms of CCN3 (reviewed in (Perbal 2001)). However, only one group has investigated the specific nature of the cleavage event. Following CCN3 overexpression in Sf9 insect cells, a secreted protein of 44 kDa was identified that was compatible with full-length CCN3. In addition, a shorter 25 kDa form was found in the insect cell conditioned media and was found to correspond to the TSP-1 and CT domain (Perbal et al. 1999). N-terminal microsequencing revealed that this CCN3 isoform resulted from a cleavage event at amino acid 187, which is located within the hinge region of CCN3. A truncated protein corresponding to this size was also reported in canine MDCK cells, HeLa human cervical cancer cells and BTK-143 human osteosarcoma cells (Perbal 1999).

Several investigators have detected the presence of a protein doublet migrating at 30 and 32 kDa in various embryonic and adult tissues as well as several cancers. Amino-truncated CCN3 variants of this size were reported in diverse biological fluids, conditioned media, cytoplasm and nucleus of several cell types [for example (Chevalier et al. 1998; Burren et al. 1999; Perbal 1999; Ellis et al. 2000; Su et al. 2001; Thomopoulos et al. 2001; Fu et al. 2004; Kyurkchiev et al. 2004; McCallum et al. 2006; Vallacchi and Rodolfo 2009)]. Finally, in brain and adrenal tissues from the adult rat, isoforms of 38–40 kDa and 18 kDa were observed and correspond to N-terminally truncated proteins. It was proposed that these isoforms are derived from proteolysis and/or glycosylation of the CCN3 protein (Su et al. 2001).

It is clear that multiple isoforms of CCN3 exist, which arise through alternative splicing or proteolytic processing. It is less clear what effect these alternative CCN3 forms have on biological responses to CCN3. Thus, it is conceivable that the relative abundance of full length CCN3 to variant CCN3 will dramatically influence how cells respond to this protein and this underexplored aspect of CCN3 biology may account for some apparent discrepancies in CCN3 action in various cell types.

## CCN3 functions in normal bone physiology

**Fig. 2:**
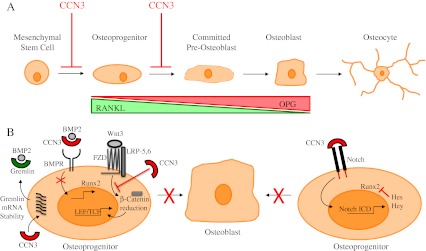
CCN3 is a physiological regulator of osteoblast differentiation. **a** Schematic depicting the different stages during osteoblast differentiation. The prevailing data suggests that CCN3 can block the differentiation of mesenchymal stem cells and/or osteoprogenitors into committed pre-osteoblasts. As mesenchymal stem cells differentiate into mature osteoblasts, Receptor Activator of NF-κB Ligand (RANKL) expression decreases and the levels of Osteoprotegerin (OPG), a decoy receptor for RANKL, increase. Thus, CCN3 would have the indirect effect of elevating the RANKL/OPG ratio in pre-osteoblasts by impairing osteoblast differentiation. **b** Mechanisms underlying CCN3-mediated blockade of osteoblast differentiation. CCN3 can directly bind and neutralize BMP-2, a potent osteoblastic factor. In addition, CCN3 can indirectly inhibit BMP2 effect by increasing the level of Gremlin, a known antagonist of BMP2, by favoring the stabilization of Gremlin mRNA. CCN3 also counteracts the signaling of Wnt3, by causing a reduction of β-Catenin levels and a subsequent diminishment of Wnt3 target genes, through a mechanism that does not interfere with the ligand-receptor interaction. CCN3 can also bind and activate Notch, causing the release the Notch intracellular domain (NICD). The NICD induces the expression of Hairy/Enhancer of Split (HES) and Hairy/Enhancer of Split with YRPW motif 1 (HEY), which in turn physically interact with and repress Runx2 activity, a key transcription factor involved in osteoblast differentiation

Of the cell types that reside in the bone microenvironment, osteoblasts are the bone forming cells and osteoclasts are responsible for resorbing bone. Mesenchymal stem cells differentiate into osteoblasts through a multi-step process (Fig. 2a) that is stimulated by BMP and Wnt family members and inhibited via Notch1 signaling (reviewed in (Krishnan et al. 2006; Canalis 2008; Long 2011)). The proper differentiation of precursors into osteoblasts requires expression of the Runx2 transcription factor, which acts as a master regulator of this process (Marie 2008).

Following stimulation with factors such as Macrophage Colony Stimulating Factor (M-CSF) and Receptor Activator of NF-kB Ligand (RANKL), hematopoietic precursors differentiate into mature activated osteoclast (Boyle et al. 2003). This process can be impaired by Osteoprotegerin (OPG), a soluble decoy receptor for RANKL. The tight control of the ratio between RANKL and OPG is critical for maintaining homeostasis of the bone (Khosla 2001; Dougall and Chaisson 2006; Ooi et al. 2011). The highest level of RANKL is secreted from osteoblast precursors and its expression decreases during osteoblast differentiation whereas OPG levels are low in osteoprogenitors and increase during osteoblastogenesis (Gori et al. 2000). In addition to RANKL and OPG, calcium signaling is critical for osteoclast differentiation, in part through its ability to induce nuclear localization of Nuclear Factor of Activated T cells c1 (NFATc1), an important transcription factor for osteoclastogenesis (Negishi-Koga and Takayanagi 2009).

Several members of the CCN family have been shown to regulate the differentiation of bone cells (Schutze et al. 2005; Kawaki et al. 2008; Su et al. 2010) (reviewed in (Yamaguchi et al. 2008; Katsube et al. 2009b; Arnott et al. 2011)). Indeed, CCN proteins are related to known antagonists of BMP proteins, such as Twisted Gastrulation (Tsg) and Chordin (Garcia Abreu et al. 2002). Until recently, a role for CCN3 in regulating the differentiation and function of bone resident cells was not known. However, the activity of CCN1 and CCN2 in these processes, coupled with the identification of certain CCN3 interacting proteins such as BMP and Notch, raised the possibility that CCN3 may also regulate bone homeostasis.

To determine whether CCN3 also plays important roles in the bone, mice overexpressing CCN3 in osteoblasts, under control of the osteocalcin promoter, were generated and characterized (Rydziel et al. 2007). Two independent transgenic lines were analyzed; the first exhibiting CCN3 expression in calvarial extracts that ranged between 25 and 250 times the levels detected in non-transgenic controls. Male and female mice, which were heterozygous for the transgene, displayed a 30–35% decrease in trabecular bone volume but exhibited similar numbers of osteoblasts, osteoclasts and similar degrees of bone erosion. However, diminished mineral apposition and bone formation was observed, which suggested impaired osteoblast function. In the second transgenic line, CCN3 mRNA expression ranged from 1,000 to 9,000-fold higher when compared to the wild type control mice. Animals from this transgenic line suffered from osteopenia, a condition characterized by significantly diminished bone mineral density. This phenotype was correlated with an increase in bone resorption and elevated osteoclast numbers along the eroded bone surfaces. These data argue that CCN3 plays a significant role in suppressing osteoblast differentiation and, at high concentrations, may enhance osteoclast differentiation or function.

This conclusion was further supported by phenotypes associated with mice bearing a targeted disruption in the CCN3 gene. These mice were engineered to express a CCN3del3 mutant, which encoded a protein lacking the VWC domain (Heath et al. 2008). While full length CCN3 could not be detected in these animals, the CCN3del3 protein was present at low but detectable levels. Interestingly, a transcript lacking the VWC domain was also present in several tissues of the wild-type embryos, raising the possibility that this isoform of CCN3 could arise naturally from alternative splicing. In CCN3del3 expressing embryos, the skeleton showed abnormalities such as enlarged vertebrae, elongated long bones and digits. In addition, joint defects such as fusions, malformations, dislocations and laxity were observed mice expressing CCN3del3. In adult animals, overgrowth of the bone and joint defects were also reported. These data argue that the normal role for CCN3 is to suppress bone formation by osteoblasts, and that the loss of CCN3 resulted in excessive bone formation. However, the authors could not definitively conclude that skeletal phenotypes observed in the CCN3del3 mice resulted from the loss of CCN3 or to a new function ascribed to the truncated CCN3del3 protein (Heath et al. 2008).

More recently, a complete knock-out of CCN3, in which exons 1, 2 and a portion of exon 3 were removed, has been described (Shimoyama et al. 2010). No overt skeletal phenotypes were reported in these CCN3^−/−^ mice; however, this study focused primarily on the role of CCN3 in the vasculature (Shimoyama et al. 2010). Another CCN3 null mouse has been developed, where the entire coding sequence of CCN3 was deleted (Canalis et al. 2010). CCN3 null mice did not demonstrate skeletal abnormalities during development and only very modest bone defects were observed with low penetrance. For example, an increase in osteoblast numbers/bone surface, mineralization and bone formation was apparent in ten-month-old females; however, this phenotype was not evident in males of the same age (Canalis et al. 2010).

Another interesting aspect of CCN action within the bone microenvironment is their potential roles in the fracture repair process. Indeed, CCN1, CCN2 and CCN4 have all been shown to be overexpressed during various phases of the fracture repair process (Hadjiargyrou et al. 2002; Nakata et al. 2002; French et al. 2004). Moreover, blocking antibodies against CCN1 disrupt fracture healing, providing evidence that CCN family members play a functional role in this process (Athanasopoulos et al. 2007). To date, no studies have implicated CCN3 in fracture repair and represent an interesting avenue of investigation that would further clarify the influence of CCN3 on bone resident cells.

Taken together, these data argue that CCN3 can influence osteoblast, and potentially osteoclast, differentiation and/or function. However, it is clear that CCN3 exerts the greatest effects on bone formation/remodeling when it is overexpressed, rather than when physiological levels of CCN3 are lost. Another important aspect when evaluating the role of CCN3 in bone is the expression level of the other CCN family members, including CCN1 and CCN2. For example, studies on CCN2^−/−^ mice revealed that CCN3 becomes overexpressed in a context of CCN2 knock-out (Kawaki et al. 2008). Considerations such as these may be important in understanding CCN3 action in bone and begin to shed light on factors that may influence the cellular context in which CCN3 can exert its biological effects.

## Mechanisms of CCN3 action in osteoblasts

The above studies argue that CCN3 regulates osteoblast differentiation. While several of these *in vivo* studies suggest that CCN3 can negatively regulate osteoblast differentiation, the mechanisms underlying CCN3 action have been revealed using *in vitro* cell culture systems. Using a MC3T3 murine calvarial derived cell model, which can be induced to differentiate into osteoblast-like cells, CCN3 was shown to inhibit osteoblast differentiation by binding to and neutralizing BMP2, a well-known enhancer of osteoblastogenesis (Minamizato et al. 2007). This led to diminished mRNA expression of several BMP2 target genes, such as Runx2, Osterix, Alkaline Phosphatase (ALP) and Osteocalcin. In addition, CCN3 was also shown to augment the release of the Notch Intracellular Domain (NICD) from full length Notch, leading to an increase in Hairy/Enhancer of Split (HES) and Hairy/Enhancer of Split with YRPW motif 1 (HEY) transcription and ultimately to impaired osteoblast differentiation (Minamizato et al. 2007).

In another study, CCN3 overexpression in ST-2 cells, a stromal cell line derived from mouse bone marrow, also resulted in a block in osteoblast differentiation (Rydziel et al. 2007). CCN3-expressing ST-2 cells displayed reduced numbers of mineralized nodules and diminished levels of Smad-1, Smad-5, and Smad-8 phosphorylation when these cells were stimulated with BMP2. In this cell system, CCN3 exerted these effects by directly binding and neutralizing BMP2 and through impairment of Wnt3 signaling (Rydziel et al. 2007). Likewise, using ST-2 and MC-3T3 cells, CCN3 was shown to impair osteoblast differentiation through BMP2 neutralization and impairment of Wnt3 signaling, in a Notch independent manner (Canalis 2007).

Yet another potential mechanism has been proposed to account for CCN3-mediated impairment of osteoblast differentiation. Overexpression of CCN3 in ST-2 cells leads to increased Gremlin expression, a known BMP2 antagonist (Gazzerro and Canalis 2006), through a mechanism involving Gremlin mRNA stabilization (Smerdel-Ramoya et al. 2011). Thus, CCN3 can function to blunt BMP2 action by directly binding and neutralizing BMP2 or through indirect mechanisms that involve stabilization of BMP2 antagonists.

CCN3 was shown to decrease proliferation and osteoblast differentiation of Kusa-A1 osteogenic mesenchymal stem cells through Notch-1 mediated p21 upregulation. This inhibitory function was also dependent on CCN3’s ability to bind directly to BMP2. These results argued that the ability of CCN3 to bind BMP2 in a CT domain independent manner is an important mechanism through which it can block osteoblast differentiation (Katsuki et al. 2008; Katsube et al. 2009a).

These findings are supported by the observation that primary calvarial bone marrow cells from CCN3^−/−^ mice revealed enhanced osteoblast differentiation in response to BMP2 when compared to cultures from wild-type controls (Canalis et al. 2010). Moreover, in both ST-2 and MC3T3 cells, siRNA-mediated reduction of CCN3 expression led to an increase in ALP expression and enhanced activity of a BMP reporter construct following BMP2 treatment. In agreement with previous results, CCN3 was shown to bind directly to BMP2. When investigating osteoclast differentiation, calvarial cultures from CCN3^−/−^ mice displayed higher numbers of osteoclasts when vitamin D3 was used as an inducer of osteoclastogenesis, raising the possibility that CCN3 suppresses osteoclast differentiation as well (Canalis et al. 2010).

Together, these data support a role for CCN3 as an inhibitor of osteoblast differentiation through multiple mechanisms, which include interfering with BMP2 and/or Wnt/β-Catenin signaling or via activation of the Notch1 pathway (Fig. 2) (Canalis 2007; Rydziel et al. 2007; Yamaguchi et al. 2008; Smerdel-Ramoya et al. 2011). It should be noted that not all studies are in agreement with a role for CCN3 in negatively regulating osteoblast differentiation. In fact, a recent study demonstrated that CCN3 favors osteoblast differentiation and bone mineralization by inducing the expression of BMP-4, a potent inducer of osteoblast differentiation. These CCN3-mediated effects were dependent on α5β1 and αvβ5 integrin receptors and subsequent activation of Integrin Linked Kinase (ILK), p38 and c-Jun Kinase (JNK) (Tan et al. 2011). Interestingly, the concentrations of CCN3 (3–30 ng/ml) that induced osteoblast differentiation were much lower when compared to CCN3 concentrations (300–600 ng/mL) that blocked osteoblast differentiation (Rydziel et al. 2007; Ouellet et al. 2011; Tan et al. 2011). These observations are reminiscent of the *in vivo* data, which revealed different effects depending on whether CCN3 was present in physiological levels or whether it was overexpressed.

## CCN3 in primary bone cancers

### Osteosarcoma

Osteosarcoma is comprised of a group of neoplasms that are usually of high grade and stage when diagnosed. They are thought to originate from mesenchymal cells having osteoblastic features. The incidence of this cancer is low in the general population although it is the most diagnosed and lethal bone cancer in young adults (Tang et al. 2008). CCN3 expression in osteosarcoma cell lines is variable. Immunohistochemistry performed on musculoskeletal tumors reveal high CCN3 expression in osteoblasts, osteoclasts, chondrocytes and skeletal muscle cells. Interestingly, primary tumors of osteosarcoma patients, who developed lung metastases, presented with high CCN3 expression (Manara et al. 2002).

Using osteosarcoma derived cell lines; CCN3 expression was found to be inversely correlated with ALP expression (Manara et al. 2002). It is known that osteosarcomas are characterized by impaired osteoblastic differentiation and generally lack markers of terminal differentiation, which has been associated with disrupted Runx2 function (Thomas et al. 2004). Interestingly, forced expression of CCN3 in normal osteoblasts can result in Notch pathway driven inhibition of Runx2 (Minamizato et al. 2007). Moreover, CCN3 has been shown to be upregulated in osteoblast-like osteosarcoma Saos-2 and OS7 cell lines (Perbal et al. 2008). Thus, it has been hypothesized that high CCN3 expression in osteosarcoma might activate Notch signaling, resulting in osteosarcoma cells being locked into an early stage of osteoblastic differentiation. In agreement with this hypothesis, CCN3 mRNA levels are increased in osteosarcoma cell lines and tumor tissues when compared to osteoblasts. Indeed, high CCN3 expression in osteosarcomas was not associated with osteoblast differentiation markers and, more importantly, correlated with shorter disease free survival (Perbal et al. 2008). This clinical correlation is in agreement with the recent observation that CCN3 can enhance the migratory properties of osteosarcoma cells, which involves αvβ5 integrin-mediated upregulation of COX-2 expression (Huang et al. 2011).

### Ewing’s sarcoma

Ewing’s sarcoma is the second most commonly diagnosed bone tumor and is found in rapidly growing bone of children (Jurgens and Dirksen 2011). While CCN3 is expressed in approximately 30% of Ewing’s sarcomas, it is associated with shorter disease free survival (Manara et al. 2002). Ewing’s sarcoma cell line (TC71) engineered to overexpress CCN3 displayed decreased cell proliferation *in vitro*, growth in soft agar and tumorigenicity when injected into mice (Benini et al. 2005). However, these CCN3-expressing cells showed increased migration and invasion using Boyden chamber assays. Increases in cell invasion were correlated with increased levels of MMP9 associated with the cell surface (Benini et al. 2005). MMP9 was previously shown to trigger the switch between the stationary and migratory state by altering cytoskeleton rearrangement through activation of RhoA/Paxillin/β-catenin signaling (Sanceau et al. 2003). In addition, overexpression of CCN3 caused a decrease in cancer cell adhesion to collagen I and collagen IV, which might be due to decreased levels of α2β1 integrin. However, no significant differences were seen in the ability of Ewing’s sarcoma cells to adhere to Laminin, Fibronectin or Vitronectin (Benini et al. 2005).

Immunohistochemistry on 170 human Ewing’s sarcoma samples revealed that CCN3 was more highly expressed in recurrences (6/8) and metastases (8/10) when compared to the primary tumor (79/152). Microarray analyses on 30 Ewing’s tumors, coupled with immunohistochemistry on 125 cases that possessed sufficient clinical data and follow-up, revealed that low levels of CCN3 expression were associated with better patient prognosis when disease free and overall survival was considered. Interestingly, domain specific antibodies revealed that 41% of the tumors expressed a protein lacking the VWC domain. Expression of this truncated protein confers a better prognosis to the patient (Perbal et al. 2009).

### Chondrosarcoma

Chondrosarcoma are malignant tumors of cartilage tissue that do not respond well to chemotherapy or radiotherapy treatment (Jamil et al. 2010). CCN3 expression in chondrosarcoma was reported to be higher in well-differentiated tumors and associated with a better patient prognosis (Manara et al. 2002; Yu et al. 2003; Benini et al. 2005). Immunohistochemical analyses revealed that infiltrates of giant-cells within chondrosarcomas exhibited strong staining of CCN3 (Manara et al. 2002). Studies on a chondrosarcoma-derived cell line showed that CCN3 increases their migration and up-regulates MMP-13 expression and activity. CCN3-induced migration was associated with engagement of the αvβ5 and αvβ3 integrins and subsequent activation of Focal Adhesion Kinase, Phosphatidylinositol 3- kinase, Akt and NF-κB signaling pathways (Tzeng et al. 2011).

These data reveal that CCN3 can play diverse roles in modulating the malignant phenotype in primary bone cancers. In osteosarcoma, CCN3 enhances the malignant phenotype of these cancer cells by limiting the extent of osteoblastic differentiation and promoting their migratory and invasive properties. Similarly, high CCN3 expression is also associated with a poor outcome in Ewing’s Sarcoma and elicits enhanced migratory and invasive responses in these cells. In contrast, CCN3 expression has been associated with a more differentiated phenotype and better prognosis in chondrosarcoma. These differences likely reflect the role played by CCN3 in the normal differentiation programs of chondrocytes and osteoblasts. CCN3 is a positive regulator of chondrocyte differentiation through the induction of TGF-β2, which is important during the condensation stage of chondrocyte differentiation (Kawai et al. 1999). Moreover, TGF-β1 functions to impair chondrocyte differentiation (Valcourt et al. 2003), and has been shown to diminish CCN3 levels (Parisi et al. 2006). In contrast, CCN3 acts as a negative regulator of osteoblast differentiation. Thus, the prognostic significance of CCN3 may be linked to its normal role during the differentiation of these bone resident cells.

## CCN3 expression in bone metastatic cancers

The vast majority of cancers affecting bone are not derived from primary bone tumors, but rather metastatic cancer cells that have spread from distant organs to colonize the bone. The two solid cancers that metastasize with the highest frequency to bone include breast and prostate cancer. Given the effects CCN3 has on the differentiation and function of bone resident cells, it may not be surprising that CCN3 has been implicated in the progression of these cancers as well.

### Breast cancer

Breast cancer is the most frequently diagnosed cancer in North American woman. Immunohistochemical staining of CCN3 in normal breast tissue, CCN3 reveals intense staining in epithelial cells, with weaker immune-positivity in the stroma and endothelium (Jiang et al. 2004). Several reports have investigated the association of CCN3 expression with clinical parameters of patients with breast cancer. In one small study, CCN3 transcript levels did not correlate with clinical parameters such as age of the patient, hormone receptor and HER2 status, grade, stage, lymph node involvement or tumor size. Only a fraction (5/44) of samples displayed higher levels of CCN3 in the tumor compared to normal breast tissue (Xie et al. 2001). In a different cohort of 122 human breast tumors and 32 normal breast tissues, lower CCN3 mRNA and protein levels were observed in tumor tissues when compared to normal samples. In addition, CCN3 expression was significantly lower in tumors of high grade in those patients diagnosed at advanced stage of the disease (Jiang et al. 2004). These studies argue that high CCN3 expression in breast cancers is associated with a better outcome.

In breast cancer-derived cell lines, low expression of CCN3 was also reported in the aggressive MDA-MB-231 human cell line when compared to the less invasive MCF-7 line (Jiang et al. 2004). Using MCF-7 cell line as a breast cancer model, CCN3 was reported to be a direct target of estrogen. Indeed, CCN3 expression was repressed in MCF-7 cells following treatment with estrogen (17-β-estradiol) (Vendrell et al. 2004). Breast cancers that express the Estrogen Receptor (ER) are classified as luminal breast cancers and stratify patients for disease management using anti-estrogen therapies such as tamoxifen (Girdler and Brotherick 2000). However, a portion of patients with ER + disease are intrinsically resistant to Tamoxifen and the majority of patients that initially respond to anti-estrogen therapies will develop resistance. Interestingly, CCN3 expression was found to be elevated in a sub-population of MCF-7 cells (MVLN) that were selected for resistance to tamoxifen or fulvestrant (Demirpence et al. 1993; Ghayad et al. 2009). Moreover, CCN3 was highly expressed in breast cancers from patients who had relapsed following tamoxifen treatment versus tumors from those that did not. In addition, CCN3 was one of five genes associated with shorter disease free survival in ER + patients treated with Tamoxifen (Ghayad et al. 2009).

As mentioned previously, the effects of CCN3 can be context dependent. Thus it has been shown that overexpression of CCN3 in MDA-MB-231 breast cancer cells results in reduced growth but has no effect on breast cancer cell adhesion to Collagen I, Collagen IV, Fibronectin or Vitronectin. Rather, CCN3 expression enhanced the ability of MDA-MB-231 cells to form spheroids in 3-D culture through increased intercellular adhesion. Interestingly, full length CCN3 was unable to induce the migration of MDA-MB-231 cells, whereas a CCN3 mutant lacking the CT domain was able to induce a migratory phenotype. These observations highlight the diversity of responses elicited by CCN3 in different cell types, even between ER + and ER- subtypes of breast cancer (Sin et al. 2009).

Relatively little is known about the role of CCN3 in metastatic breast cancer. The first hint that CCN3 may play a role in organ-specific metastasis came from the analysis of gene expression profiles derived from 58 breast cancer metastases. Interestingly, CCN3 was found to be highly expressed in the majority of the bone (16 samples) metastases but was detected at very low levels in metastases derived from lung (18 samples), brain (19 samples) and liver (5 samples) (Zhang et al. 2009).

**Fig. 3:**
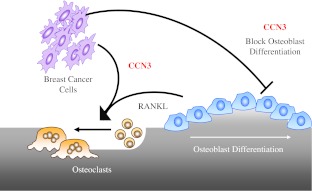
Potential roles for CCN3 in promoting the formation of osteolytic breast cancer metastases. CCN3 expressed by breast cancer cells can block the differentiation of mature osteoblasts, utilizing the mechanisms outlined in Fig. 2. This would result in a tip towards higher levels of Receptor Activator of NF-κB Ligand (RANKL) produced by pre-osteoblasts and less Osteoprotegerin (OPG). The higher levels of RANKL would serve to induce the differentiation of monocytes into osteoclasts. In concert with its negative effects on osteoblast differentiation, CCN3 can also sensitize osteoclast precursors to respond to RANKL, enhancing osteoclastogenesis and bone resorption

In our own work, we also have shown that CCN3 is expressed in bone metastasis samples from patients with breast cancer and have identified CCN3 as a gene highly expressed at the mRNA and protein levels in 4T1 murine breast cancer cells selected for their ability to metastasize to bone (Rose et al. 2007; Ouellet et al. 2011). We also demonstrated that CCN3 enhanced the bone metastatic ability of 66cl4 cells, which are known only to metastasize to the lungs, without altering their growth in the mammary fat pad (Ouellet et al. 2011). Our interest in CCN3 as a factor that could promote the formation of osteolytic bone metastases stemmed from its demonstrated roles in regulating the differentiation and activity of bone resident cells, as discussed above. During the formation of osteolytic lesions, the balance between bone formation and resorption is tipped in favor osteoclast differentiation and bone destruction. In agreement with previous studies, CCN3 was able to impair osteoblast differentiation from primary bone marrow cultures, resulting in higher RANKL/OPG ratios which would enhance osteoclastogenesis. Furthermore, we demonstrated that CCN3 promotes osteoclast differentiation from RANKL-primed monocyte precursors (RAW264.7) through a mechanism involving calcium oscillations and NFATc1 nuclear translocation (Ouellet et al. 2011). Interestingly, the ability of CCN3 to promote the formation of osteolytic bone metastases may reflect the fact that breast cancer cells colonizing the bone would contribute to significantly elevated levels of CCN3, which would tip the balance towards osteoclastogenesis (Fig. 3).

Recently, it has been argued that the expression level of CCN3 alone may not be the most informative, but rather the ratio of CCN3 versus CCN1 or CCN2 is more important. High CCN1/CCN3 or CCN2/CCN3 mRNA ratios were found to be associated with a highly metastatic phenotype in breast cancer cells. In addition, high expression of CCN2 relative to low CCN3 levels was associated with enhanced tumor formation, angiogenesis and the ability to form bone metastases (Ohgawara et al. 2011). This is in contrast to our findings in which we observed high CCN3 levels in the context of low CCN1 and CCN2 expression in bone metastatic breast cancer cells (Ouellet et al. 2011) and also contrasts with the observation that human breast cancer bone metastases that display high CCN3 mRNA expression (Zhang et al. 2009). One potential explanation for these differences stems from the fact that we analyzed expression of CCN family members in breast cancer cells that had formed bone metastases and were explanted back into culture (Ouellet et al. 2011); whereas, CCN1/CCN3 and CCN2/CCN3 ratios in the MDA-MB-231 model were determined in unselected parental breast cancer cells that had not formed bone metastases (Ohgawara et al. 2011). The possibility that CCN3 expression is selected for in bone metastases is substantiated by the detection of CCN3 specifically in bone metastases (Zhang et al. 2009). Indeed, the notion that, following cancer cell selection and isolation from distinct metastatic sites, the expression of genes in the selected populations can vary dramatically from the starting cell population is now widely accepted (Kang et al. 2003; Minn et al. 2005; Rose et al. 2007; Bos et al. 2009; Tabaries et al. 2011). The data supporting a high CCN2/CCN3 ratio in the bone metastases was based solely on immunohistochemical staining (Ohgawara et al. 2011). However, immunohistochemical staining in bone metastases derived from MDA-MB-231 cells cannot be used to make such quantitative arguments regarding the relative ratio of CCN2 to CCN3, given that these antibodies will undoubtedly have very different affinities for their respect targets (Ohgawara et al. 2011).

### Prostate cancer

Relatively little is known concerning the role of CCN3 in prostate cancer progression and metastasis. Only one study has examined CCN3 levels in prostate cancer cell lines and tissues, which revealed that CCN3 expression was higher in prostate cancer-derived cell lines compared to immortalized prostate epithelial cells (Maillard et al. 2001). It is intriguing to note that the highest level of CCN3 expression was observed in the PC3 prostate cancer cell line, which forms aggressive osteolytic bone metastases when injected into mice (Angelucci et al. 2004). Furthermore, 19 of 20 prostate adenocarcinoma samples stained positively for CCN3 expression by immunohistochemistry. However, the sample size was too small to make meaningful correlations between the intensity of CCN3 staining and Gleason score (Maillard et al. 2001). The established role of CCN3 as an inhibitor of osteoblast differentiation (Canalis 2007; Minamizato et al. 2007; Rydziel et al. 2007; Katsuki et al. 2008; Yamaguchi et al. 2008; Katsube et al. 2009a; Canalis et al. 2010; Ouellet et al. 2011), coupled with the recent observation that low CCN3 concentrations may enhance osteoblastogenesis, raises the intriguing possibility that CCN3 could play a role in lytic and/or blastic bone metastases formation depending on the level of CCN3 expression. However, this will require further functional validation to determine whether CCN3 plays a functional role in prostate cancer growth and metastasis to bone.

## Conclusion

CCN3 exerts numerous effects on both normal and transformed cells, and the cellular responses can be very different depending on the various CCN3 isoforms and the cellular context in which CCN3 is acting. Moreover, increasing data suggests that the ultimate outcome of cellular differentiation that is regulated by CCN3 may also depend on the level of CCN3 expression. Available data points toward an important role for CCN3 in the negative regulation of osteoblast differentiation, which has clear consequences for normal bone homeostasis and turnover. In addition, CCN3 has been implicated as a poor prognostic marker in several primary bone cancers, including osteosarcoma and Ewing’s sarcoma. Given the ability of CCN3 to modulate the differentiation status and activity of bone resident cells, it is interesting to note that breast cancer cells that metastasize to bone and induce bone destruction select for high levels of CCN3 expression. Future studies will be needed to focus on the modular domains within CCN3 that are important for the pro-bone metastatic activities of CCN3. Moreover, very little is known regarding the role of CCN3 in prostate cancer progression and subsequent metastasis to bone.

These areas represent fertile ground for future experimentation. What is clear is that CCN3 can play complex roles in modulating cancer phenotypes, such as metastasis, as it does in the regulation of normal cellular behavior. However, there is growing evidence that CCN3 can influence the progression of primary bone cancers and act as an important pro-metastatic factor in numerous cancers, including metastatic cancers affecting bone.

## Abbreviations

ALP: Alkaline phosphatase
BMP: Bone morphogenetic protein
CT: Carboxy-terminal region
ER: Estrogen receptor
HSPG: Heparan sulfate proteoglycans
IGF: Insulin-like growth factor
IGFBP: Insulin-like growth factor binding protein
ILK: Integrin-linked kinase
JNK: c-Jun kinase
LRP: Low-density lipoprotein receptor-related protein
MAV: Myeloblastosis-associated virus
M-CSF: Macrophage colony stimulating factor
MMP: Matrix metalloprotease
NFATc1: Nuclear factor of activated T cells c1
OPG: Osteoprotegerin
RANKL: Receptor activator of NF-κB ligand
TGF-β: Transforming growth factor beta
TSG: Twisted gastrulation
TSP-1: Thrombospondin-1
VEGF: Vascular endothelial growth factor
VWC: von Willebrand factor type C domain
WISP: Wint-1 inducible signaling pathway protein
